# Colorectal perforation in cytomegalovirus colitis during chemotherapy for metastatic colorectal cancer: a case report

**DOI:** 10.1097/RC9.0000000000000629

**Published:** 2026-07-02

**Authors:** Hiroaki Shimada, Mizunori Yaegashi, Akira Sasaki

**Affiliations:** Department of Surgery, School of Medicine, Iwate Medical University, Yahaba, Japan

**Keywords:** case report, cytomegalovirus, cytomegalovirus colitis, metastatic colorectal cancer, opportunistic infections

## Abstract

**Introduction:**

Cytomegalovirus (CMV) infections often occur as opportunistic infections among immunocompromised hosts. We report a case of CMV colitis that resulted in colonic perforation in a patient undergoing chemotherapy for more than 2 years for metastatic colorectal cancer (mCRC).

**Presentation of case:**

A 61-year-old man receiving long-term chemotherapy for mCRC presented with persistent diarrhea and anorexia. Colonoscopy revealed ulcerating colorectal lesions. Following admission, he exhibited signs of peritoneal irritation and bloody stools. Computed tomography demonstrated intraperitoneal free air, prompting an emergency operation. Considering the perforations in the descending colon, we performed Hartmann’s operation. However, in addition to the histopathological results, bloody stools persisted postoperatively; thus, enteritis caused by CMV infection was suspected. Furthermore, CMV pp65 antigen-positive blood cells were detected, leading to the diagnosis of CMV colitis. Consequently, ganciclovir was administered, and persistent bloody stools resolved.

**Discussion:**

CMV infection occurs asymptomatically during childhood and then remains latent in the body over time. Although CMV colitis is common in patients undergoing chemotherapy for hematologic malignancies, it is rare in mCRC cases. However, immunocompromised patients receiving long-term steroid therapy for chemotherapy are at a high risk of CMV reactivation. Therefore, CMV colitis should be considered one of the differential diagnoses based on persistent diarrhea and bloody stools, as well as ulcer formation observed on colonoscopy.

**Conclusion:**

Although CMV enteritis during colorectal cancer chemotherapy is rare, CMV colitis should be suspected if patients experience prolonged diarrhea or bloody stools during chemotherapy. Therefore, early diagnosis by detecting CMV antigen-positive blood cells is important.

## Introduction

Colorectal cancer (CRC) is the third most common cancer worldwide^[^1^]^, and many patients with metastatic CRC (mCRC) undergo long-term chemotherapy. Cytomegalovirus (CMV) is known to cause opportunistic infections in immunocompromised patients, such as those with recent transplantation, autoimmune diseases, or acquired immunodeficiency syndrome (AIDS)^[^2,3^]^. A prolonged immunocompromised state leads to CMV reactivation, which can cause intestinal mucosal damage. Reports of this condition occurring during chemotherapy in patients with mCRC are extremely rare. However, intestinal perforation associated with CMV colitis is considered a major complication. Therefore, the early detection of CMV infection is important. This case report was prepared according to the Surgical CAse REport (SCARE) criteria^[^4^]^.

HIGHLIGHTS

- Cytomegalovirus (CMV) exposure is common, but the risk of CMV disease is high in the immunocompromised.
- CMV enteritis during colorectal cancer chemotherapy is rare.
- Protracted diarrhea or bloody stools during chemotherapy suggest CMV colitis.
- CMV pp65 antigen-positive blood cell detection helps distinguish preexisting from active CMV infection.
- Such detection is also useful for evaluating anti-CMV drug treatments.

## Presentation of case

**Figure 1.**
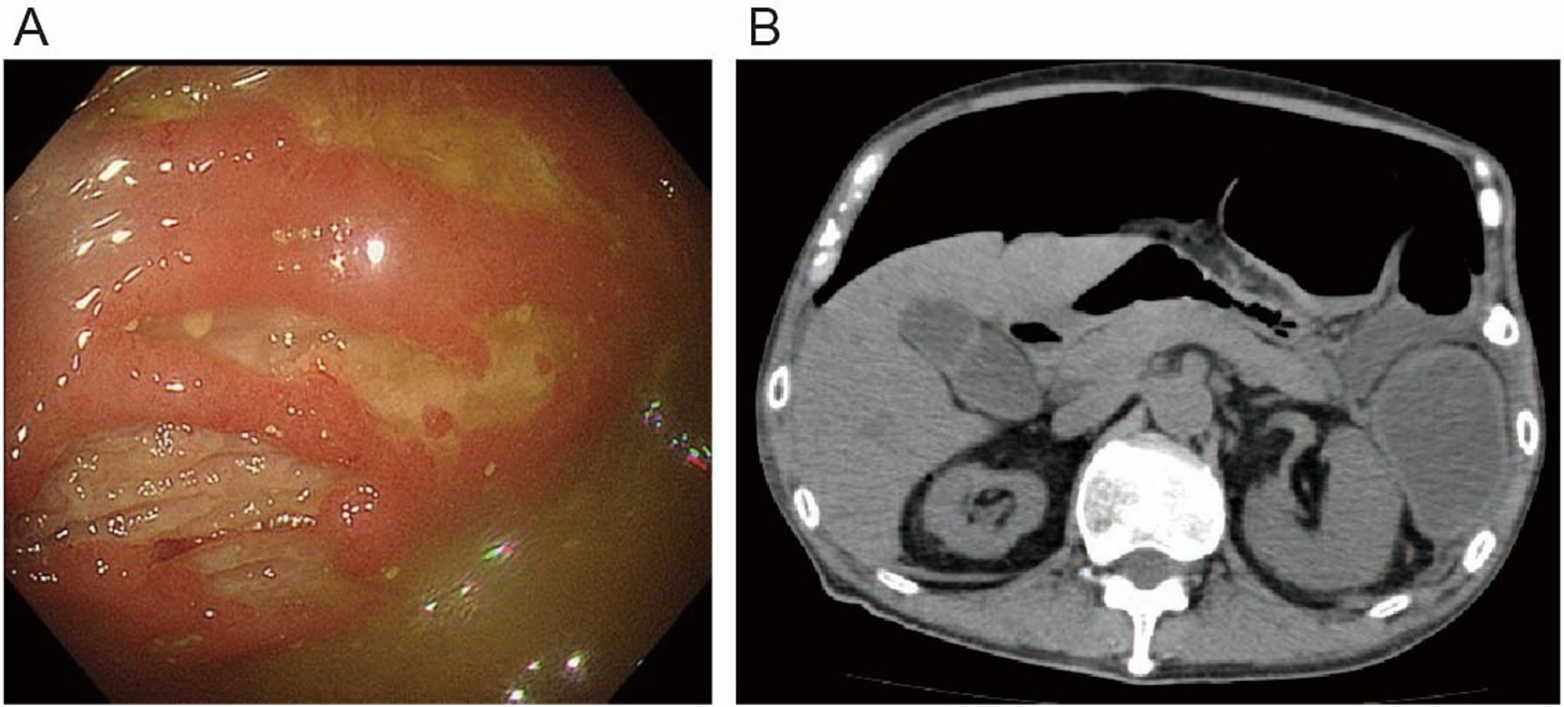
Colonoscopy and computed tomography findings. (A) Colonoscopy showing ulcerating lesions in the descending colon. (B) Abdominal computed tomography showing free air and ascites.

A 61-year-old man who underwent long-term chemotherapy for recurrent colon cancer presented with persistent diarrhea and anorexia. Four years earlier, he had undergone a low anterior resection for a rectal tumor. A histological examination of the resected specimen revealed adenocarcinoma (Ra, T4a, N1b, M0, pStage IIIa; TNM Classification of Malignant Tumors, 8th edition, Union for International Cancer Control). The patient subsequently received adjuvant chemotherapy with uracil/tegafur and leucovorin. Ten months after the first operation, computed tomography (CT) detected lung metastasis. Therefore, 8 cycles of leucovorin, fluorouracil, and oxaliplatin (mFOLFOX6) plus bevacizumab and 12 cycles of leucovorin, 5-fluorouracil (sLV5FU2) plus bevacizumab for oxaliplatin-induced peripheral neuropathy were administered. After CT confirmed disease progression, the chemotherapy regimen was changed to 12 cycles of leucovorin, fluorouracil, and irinotecan (FOLFIRI) plus panitumumab. As described previously, the patient received chemotherapy using multiple regimens over a period of approximately 2 years. During this time, steroids were administered for purposes such as antiemesis, and the total dose, expressed as prednisolone equivalents, exceeded 2000 mg. Fourteen days after his last chemotherapy, the patient presented with persistent diarrhea and appetite loss and was admitted. Seven days after admission, he developed bloody stools. Three days later, physical abdominal examination revealed severe distention and rebound tenderness. Laboratory findings showed elevated C-reactive protein and procalcitonin levels (Table 1). Colonoscopy and CT scan were performed on the same day. Colonoscopy revealed multiple irregular ulcerative lesions extending from the sigmoid colon to the rectum. A biopsy was not performed due to bleeding risk (Fig. 1A). CT demonstrated intraperitoneal free air, indicating colorectal perforation (Fig. 1B), which prompted an emergency operation. As the perforation was in the descending colon, a Hartmann’s procedure was performed. Additionally, doripenem (0.5 g thrice daily) was administered for acute peritonitis. However, bloody stools persisted after the surgery. Serology revealed a high level of serum titer for CMV immunoglobulin G and CMV pp65 antigen-positive blood cells (Table 2). Based on these clinical findings and test results, CMV colitis, in addition to severe intestinal mucosal damage caused by long-term chemotherapy, was suspected. Ganciclovir (5 mg/kg twice daily) was administered for CMV colitis 7 days after the procedure.

**Table 1:** Laboratory findings (preoperative data).

Laboratory findings			
WBC	5200/μL	CRP	16.92 mg/dL
RBC	357 × 10^4^/μL	Na	143 mmol/L
Hb	9.4 g/dL	K	3.3 mmol/L
Ht	28.30%	Cl	107 mmol/L
Plt	16.6 × 10^4^/μL	AST	27 IU/L
Neut	4620/μL	ALT	40 IU/L
Lymph	440/μL	LDH	432 IU/L
Mono	90/μL	CK	976 IU/L
Eosin	20/μL	T-Bil	0.6 mg/dL
Baso	10/μL	TP	4.7 g/dL
β-D-glucan	12.3 pg/mL	BUN	23.8 mg/dL
Procalcitonin	11.0 ng/mL	Cre	1.29 mg/dL

**Table 2:** Cytomegalovirus status.

Cytomegalovirus status	
CMV IgG-EIA^*^	44.1 U/mL
CMV C-7HRP^**^	Positive

*Enzyme immunoassay.

**A hoeseradish peroxidase-based immunoassay for CMV pp65 antigenemia.

**Figure 2.**
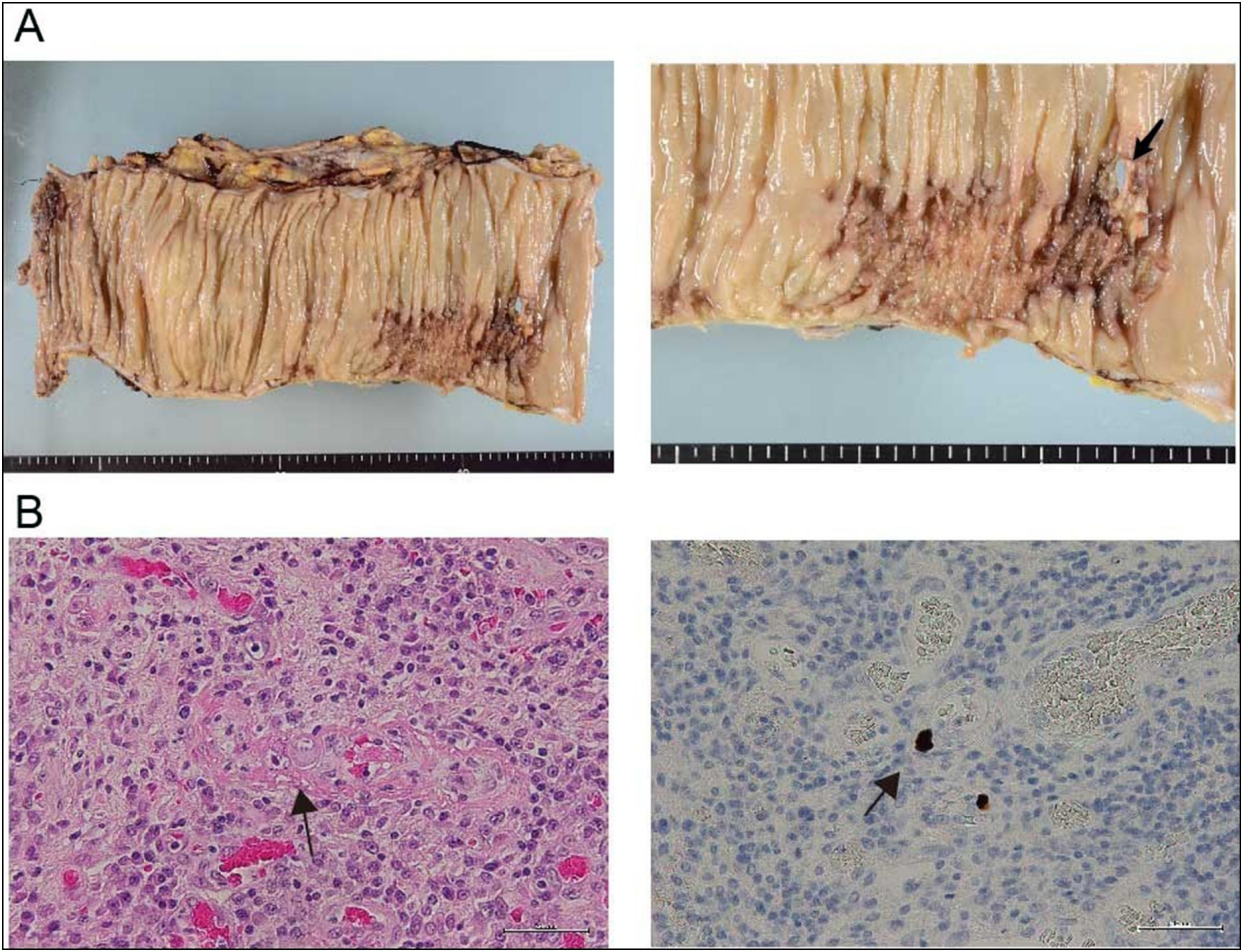
Histopathological findings. (A) Histopathological examination revealed an ulcer and a perforation in the descending colon. (B) Inclusion bodies in the ulcer (left) (hematoxylin–eosin staining, 400× magnification) and positive immunoreactivity for cytomegalovirus (CMV, right) (anti-CMV monoclonal immunostaining, 400× magnification).

Pathologically, there was an irregularly shaped ulcer in the descending colon, with a perforation at the base of the ulcer. Furthermore, viral inclusions were found in the stroma and the vascular endothelial cells of the descending colon mucosa, and CMV immunoreactivity was positive (Fig. 2). Based on these findings, the perforation was caused by an ulcer attributed to CMV colitis. On the 14th postoperative day, the CMV pp65 antigen-positive cells had become undetectable. Consequently, maintenance therapy was administered at a dose of 2.5 mg/kg twice daily for 10 days. On day 48 post-surgery, the bloody stools had resolved, and the CMV pp65 antigen-positive blood cells remained undetectable. The patient was discharged 58 days after the operation. Unfortunately, 4 months after discharge, he died from mCRC progression.

## Discussion

CMV infection occurs asymptomatically during childhood and generally remains latent in the body^[^5^]^. Many people have CMV antibodies; among the Japanese, approximately 70% have CMV antibodies^[^6^].^ CMV commonly causes opportunistic infections in immunocompromised patients, such as those with AIDS, cancer, organ transplants, or those undergoing steroid or immunosuppressive therapy. Although CMV colitis often develops during chemotherapy in patients with hematologic malignancies^[^7^],^ reports of patients receiving chemotherapy for mCRC, particularly those with colorectal perforation, are limited^[^8^]^ The clinical symptoms of CMV colitis are indistinguishable from those of irinotecan-induced enteritis, another infrequent but significant cause of severe colitis^[^9,10^]^. In our patient’s case, colitis seemed responsible for the mucosal disorder, given that he had undergone long-term chemotherapy with irinotecan. However, considering the CMV positivity in the serum and tissue and the improvement in symptoms after ganciclovir therapy, CMV reactivation due to immunosuppression from long-term chemotherapy could be the cause of CMV colitis development. Regarding the organ-specific symptoms of CMV infection, the most commonly affected is the digestive system (gastroenteritis; 31%), followed by the central nervous system (meningitis, encephalitis, and myelitis; 19%), the hematologic system (hemolytic anemia and thrombocytopenia; 7%), the vascular system (venous thrombosis and pulmonary thromboembolism; 7%), and the ophthalmologic system (uveitis and keratitis; 6%). Gastrointestinal symptoms such as diarrhea and bleeding require consideration of CMV infection testing. In CMV enteritis, the endothelial cells of the intestinal mucosa are infected, causing vasculitis and resulting in impaired mucosal blood flow. These ischemic changes can lead to ulcer formation and, in severe cases, colorectal perforation^[^11^]^. Endoscopic findings typically show punched-out ulcers. However, various ulcers can present at the same time, including shallow irregular ulcers, annular ulcers, band ulcers, longitudinal ulcers, and aphthous ulcers, without a consistent pattern. Pathological findings include granulation in the lamina propria of the mucosa, marked lymphocytic infiltration, and inclusion bodies within the nuclei of vascular endothelial cells in the lamina propria and submucosa. Based on the persistent postoperative bloody stools and histopathological examination results^[^12^]^, we suspected CMV-induced enteritis infection. To prevent major complications such as colorectal perforation, it is important to consider CMV colitis as a possible diagnosis upon the onset of persistent diarrhea or bloody stools, or if ulcers are observed during endoscopy, and to initiate antiviral therapy.

Patients on long-term steroid therapy are highly susceptible to infections^[^13^]^. This risk increases when prednisolone is administered at 20–40 mg/day or more for 4–6 weeks or longer^[^14^]^. However, infection rates do not increase for patients receiving less than 10 mg/day or a cumulative dose below 700 mg^[^15^]^. Our patient was at high risk of infection because of the high cumulative dose of prednisolone administered over 2 years (i.e., >2000 mg).

The CMV antigenemia test for CMV-emia demonstrates high sensitivity and specificity (93.5% and 94.3%, respectively)^[^16,17^],^ but the rate is lower (54%) for intestinal CMV infection^[^12^]^. Furthermore, if polymorphonuclear leukocytes (neutrophils) in the peripheral blood are few, they cannot be measured, reducing the test’s sensitivity. Therefore, there are limitations when relying on this method alone for diagnosis, and a comprehensive evaluation, including histological confirmation, is essential. Detection of CMV pp65 antigen-positive blood cells in plasma suggests active infection and corresponds to the period of clinical symptoms. Therefore, the CMV antigen plasma test may be useful for distinguishing between a pre-existing and an active CMV^[^18^]^ and evaluating the effectiveness of anti-CMV drug treatments^[^19^]^. In the present case, the CMV antigen-positive blood cells turned negative after ganciclovir therapy, and the CMV symptoms subsequently improved.

## Conclusion

Here, we report a case of CMV colitis complicated by colorectal perforation in a patient receiving chemotherapy for mCRC. Patients managed with prolonged chemotherapy may be immunocompromised, and CMV colitis should be considered if persistent gastrointestinal symptoms, such as diarrhea and bloody stools, are observed. Early clinical suspicion and prompt initiation of antiviral therapy are essential to prevent severe complications.

## Ethical approval

The anonymised presentation of this case report did not require ethical approval according to the standards of Iwate Medical University Hospital. This study was conducted according to the principles of the Declaration of Helsinki.

## Data Availability

Data sharing is not applicable to this article as no datasets were generated or analyzed during the current study.
